# Nature Prescriptions for Health: A Review of Evidence and Research Opportunities

**DOI:** 10.3390/ijerph17124213

**Published:** 2020-06-12

**Authors:** Michelle C. Kondo, Kehinde O. Oyekanmi, Allison Gibson, Eugenia C. South, Jason Bocarro, J. Aaron Hipp

**Affiliations:** 1 USDA-Forest Service, Northern Research Station, 100 North 20th Street, Ste 205, Philadelphia, PA 19103, USA; kehinde.oyekanmi@pennmedicine.upenn.edu; 2 Department of Emergency Medicine, Perelman School of Medicine at the University of Pennsylvania, Philadelphia, PA 19104, USA; eugenia.south@uphs.upenn.edu; 3 Schuylkill Center for Environmental Education, 8480 Hagys Mill Rd, Philadelphia, PA 19128, USA; apgibson514@gmail.com; 4 Department of Parks, Recreation & Tourism Management, College of Natural Resources, Box 8004, 3028F Biltmore Hall, North Carolina State University, Raleigh, NC 27695-8004, USA; jnbocarr@ncsu.edu (J.B.); jahipp@ncsu.edu (J.A.H.)

**Keywords:** nature prescriptions, NatureRx, ParksRx, narrative review, outdoor recreation

## Abstract

Nature prescription programs have emerged to address the high burden of chronic disease and increasingly sedentary and screen-based lifestyles. This study examines the base of evidence regarding such programs. We conducted a narrative review of published literature using four electronic databases. We included case studies, research design articles, and empirical studies that discussed any type of outdoor exposure or activities initiated by a health-care provider from an outpatient clinic. We examined articles for information on target populations, health outcomes, and structural and procedural elements. We also summarized evidence of the effectiveness of nature prescription programs, and discussed needs and challenges for both practice and research. Eleven studies, including eight empirical studies, have evaluated nature prescription programs with either structured or unstructured formats, referring patients either to nearby parks or to formal outdoor activity programs. Empirical studies evaluate a wide variety of health behaviors and outcomes among the most at-risk children and families. Research is too sparse to draw patterns in health outcome responses. Studies largely tested program structures to increase adherence, or patient follow-through, however findings were mixed. Three published studies explore providers’ perspectives. More research is necessary to understand how to measure and increase patient adherence, short and long-term health outcomes for patients and their families, and determinants of provider participation and participation impacts on providers’ own health.

## 1. Introduction

The chronic disease burden in the U.S. is a significant cause of concern. Forty percent of the population has two or more chronic conditions such as hypertension, diabetes, and mood disorders [1]. One hundred and seven million Americans are obese and over 16 million adults have had an episode of depression each year [2,3]. Among children, the prevalence of chronic disease has doubled from 12.8% to almost 27% since the 1990s [4]. Faced with this burden, children today are moving into adulthood with increasingly complex medical problems and needs [5,6].

Many health behaviors during childhood contribute to the development of chronic disease in adulthood. For example, children have increasingly sedentary lifestyles—the average American child spends nearly eight hours a day watching a screen [7,8]. Sedentary behaviors are associated with many negative health behaviors and outcomes, including all-cause and cardiovascular disease (CVD) mortality [9]. The relationship between sedentary behaviors and poor health outcomes is strongest amongst low-income urban children [10,11,12]. For these children, neighborhood safety concerns can reduce their ability to leave their homes, resulting in higher rates of obesity. Often paired with sedentary behaviors is a lack of adequate physical activity. Less than a quarter of youths meet the American Academy of Pediatrics (AAP) guidelines for 60 min of physical activity at least five days per week [13].

Nature prescription programs aim to address the high burden of chronic disease and increase physical activity [14]. No standard definition of a nature-prescription exists, however an overview and recent history are given by James et al. [14] Nature prescriptions generally involve a physician, or other healthcare provider, giving patients a written recommendation to spend time outside. There are between 75–100 nature prescription programs across the U.S. [15]. These programs are also motivated by a growing body of research demonstrating the health benefits of spending time in nature [16,17].

Nature as a health promotion tool is garnering wider institutional support in the U.S. The AAP named connecting children and families with nature a top priority area for 2019 [18]. The recent U.S. National Physical Activity Plan [19] recommended the use of park prescriptions. Nature prescriptions are attractive because they leverage existing services such as parks and outdoor programs to facilitate sustained involvement in healthy behavior.

Previous reviews have focused on the health impacts of “nature-assisted” [20] gardening [21] and horticultural therapies [22], and green exercise [23,24,25]. While all of these therapies involve the connection between nature and human health, they each do so in different ways. The predominant nature-assisted therapies that have previously been reviewed, such as horticultural and wilderness programs, or green exercise, have either lacked a clinical component [26,27,28], or have been in-patient programs [29,30]. Studies that have lacked a clinical component have evaluated outcomes of participation in the program or activity itself. For example, study participants are directly recruited from program participants, which is most often the case with wilderness programs (e.g., see Hattie et al. [31]) Studies of green exercise reviewed most recently by Mnich et al. [25] also predominantly lack a clinical component. Other studies have assessed outcomes of in-patient programs and very frequently horticultural programs in which institutionalized patients are referred to in-house activities (e.g., see Annerstedt and Währborg (2011)). Although these programs and interventions all involve human–nature interaction and measurements of effects on health, some of them lack the involvement of the medical provider and medical institutions. Medical providers are the fundamental institution involved in maintaining the health and well-being of society at large, and could pave the road toward wide-scale re-involvement of natural amenities in health care. On the other end of the scale, nature therapy research that focuses only on in-patient settings lack an element of applicability to the general public.

We therefore focus our review on studies of nature prescription programs that involve a clinical component in out-patient settings. We conducted a narrative review of existing literature, with the aim of interpretation and critique [32], on nature prescriptions. The questions that we address with our review are: (1) What populations and health outcomes are targeted? (2) What structural and procedural elements make up a nature prescription program? What program components, settings, leadership aspects, partnership needs and funding sources are described? (3) What enables program adherence? (4) What nature prescription health impacts, and providers’ needs, have been documented in empirical studies? After exploring these questions, we discuss needs and challenges for both practice and research.

## 2. Materials and Methods

To address our research questions, we searched electronic databases in June 2019, including: Web of Science, Scopus, EMBASE, and MEDLINE. We did not restrict searches by date of publication. We submitted a standard Boolean search phrase, with syntax tailored to each database such as the following in Web of Science: (“outdoor activity prescription*” OR “nature prescription*” OR “park prescription*” OR “outdoor prescription*” OR “prescrib* nature” OR “outdoor play” OR “nature based activit*” OR “outdoor physical activity” OR “nature engagement” OR “green prescription*” OR “outdoor prescription*” OR “green exercise” OR “nature play” OR “nature AND outdoor activit*” OR “green care” OR “wander garden therap*” OR “ecotherapy*” OR “horticultural therap*” OR “nature assisted therap*”). We considered only published work, and not grey literature or expert testimony, and therefore consider our review to be narrative in nature [32]. Search terms for the remaining databases are shown in the Supplementary Materials.

We included case studies, research design articles, and empirical studies conducted anywhere in the world, that discussed any type of outdoor exposure or nature-related activities initiated by a physician or other health-care provider from an outpatient clinic. We also included articles that addressed patients’, families’, or providers’ perspectives or experiences in nature prescription programs and articles that discussed other aspects of park prescription programs. We included studies of participants of all age categories.

We excluded articles about nature exposure that did not involve clinic-based physician prescriptions; articles about in-patient treatments for institutionalized individuals; articles about prescriptions for physical activity, exercise, or play without specification that the activity be done outdoors or in nature; general articles about the health benefits of nature; nature-based therapy articles; opinion pieces; and articles that were not in English.

There is some overlap in the terms social prescriptions and nature prescriptions. Social prescribing links patients with social activities to address a wide range of social and health problems [33]. In many cases, these include outdoor or nature-based activities. We included social prescriptions articles only if such prescriptions included nature-based activities.

**Figure 1:**
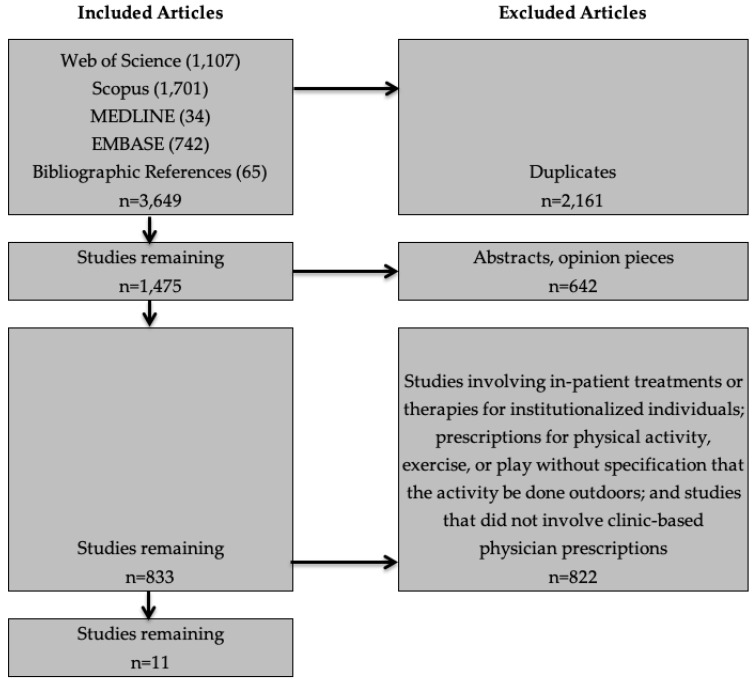
Article selection process.

Figure 1 shows the article selection process. The initial searches identified 3649 references. We used consensus among all authors to determine study inclusion and exclusion criteria. We first excluded duplicates, leaving 1475 studies. We then removed abstracts and opinion pieces, leaving 833 articles. One author (KO) reviewed the titles and abstracts of the remaining 833 articles, applying the exclusion criteria described above. One author (MK) reviewed the study inclusion/exclusion decisions, and the remaining authors reviewed conflicts or uncertain decisions.

From each study or article, we recorded the type of article (empirical study, case study, research design), research aims, study design, location, definition of nature prescriptions and program procedures, sample size, population demographics, intervention, measurement procedure and instruments, and target health outcomes. The study team assembled a template with information fields, and a single author (KO) extracted data from each of the papers in tabular format.

## 3. Results

We found 11 papers that met our inclusion criteria, including one case study [34], two research protocols or study designs [35,36], and eight empirical studies [37,38,39,40,41,42,43,44]. Two of the papers communicate research design [35] and results [44] of the same study. From these papers, we found documented nature prescription definitions, shown in Table 1. One study [38] surveyed participants from multiple programs, and did not specify program descriptions and is not included in this table.

### 3.1. Target Populations and Outcomes

Characteristics of reviewed studies are shown in Table 2. Although we did not restrict our search geographically, all studies that met our criteria were conducted in the U.S. With one exception [39], clinical programs described in our selected articles targeted children and/or their parents. More than half of the programs in our study targeted financially disadvantaged [34,35,36,41,42,43,44], minority and immigrant children [36,41,42,44]. Some of these programs targeted infants and toddlers [34,41]. Parents and families were often invited to participate in the prescribed outdoor activities [35,41,42,44]. Two programs targeted children at risk of chronic disease [36,43], including overweight or obesity status, high blood pressure, family history of diabetes and/or CVD. Five studies specified an urban population [34,36,41,42,44], while two studies focused on rural populations [37,40].

Health-related outcomes or behaviors measured varied widely. A set of studies measured providers’ views of nature [40], perspectives [38,41], and behaviors [40] associated with nature prescriptions. Another set of studies measured patient (or care-giver) adherence (whether or not they spent prescribed time in nature, and how much time) to the nature prescription [38,39,40,42,43,44], attentional fatigue and performance [39], sedentary time [38], loneliness [35], nature affinity [35], stress [42,44], resilience [42], physical activity [36,44], body mass index (BMI), blood pressure, self-esteem, social anxiety, and quality of life [36].

### 3.2. Structural and Procedural Elements

#### 3.2.1. Structured Versus Unstructured Program Components

We found two distinct types of nature prescriptions. Structured prescriptions involved patient counseling and referral to a formal outdoor program or activity. Unstructured programs generally involved clinical counseling and education about nearby outdoor resources.

Structured prescriptions instructed patients to participate in nature walks [35,41,44], outdoor sports [36,41], outdoor games [35,44], picnics [35,44], unstructured outdoor play [35,41,44], quiet reflection [35,44], or unspecified group outings [42]. One outdoor exercise intervention was part of a comprehensive health intervention that also included education on wellness and nutrition [36]. Unstructured programs referred patients to specified locations such as local parks. In some cases, the prescription came with an incentive such as a one-day free pass to a state park [40], or other supportive items such as a journal and pedometer [35,44].

Structured programs varied in time intensity. Patients were referred to outdoor programs that occurred weekly [35,41,42,44], or daily [36]. Razani et al. [35,42,44] discussed three discrete weekly sessions for supported groups. Two programs specified the frequency and duration [37,43], as well as intensity of outdoor activity [43].

#### 3.2.2. Setting

Study recruitment occurred in various settings. Four studies reported that patients were recruited from pediatric offices [36,37,40,42]. In two studies [41,43], patients were recruited from community health centers that serviced low-income residents. Cimprich and Ronis (2003) recruited breast cancer patients from a university medical center. Four studies [34,35,42,44] conducted recruitment at a federally qualified health center. In all of these studies, healthcare providers (physicians) delivered the nature prescription during doctor visits.

#### 3.2.3. Program Leadership, Implementation, and Partnerships

Physician leaders, or “clinician champions,” were important to program function [41]. This role has been described as “faculty champions” [45], “nature champions” [45], or “general practitioner champions” [46,47]. This individual plays a leadership role in promoting and implementing programs, and in mitigating challenges. Champions promote clinician program engagement and facilitate communication between providers and program staff [41,46,47]. Champions can ensure long-term engagement [46,47], and address challenges that emerge [46]. The clinician champion may also play an important role in carrying out research and evaluation [41].

Follow-up, or case management by a non-clinical team member or coordinator was a component of a number of studies. Several studies discussed the role of a third party (e.g., study team member, clinic staff member or nurse practitioner) in the counseling of patients in order to increase prescription adherence. This type of counseling is often useful immediately following dispensation of the prescription. For example, the role of a “link worker,” responsible for connecting patients to relevant services, has been described by various authors [33,47,48].

Several studies emphasized the importance of naturalists or outdoor educators in carrying-out structured prescriptions. In one study, park staff led nature outings and monitored safety [35,44], and in another study an experienced naturalist co-facilitated (with a physician) monthly park excursions [34]. State park employees collected and compiled program admission passes, and sent them to research staff [40]. Another study suggested that park and recreation staff could conduct patient follow-up to alleviate the workload of health care providers [37].

Third parties, such as nonprofit groups, often deliver education materials to nature prescription providers detailing the benefits of nature for human health. This communication is generally tailored to the sociocultural or geographic location of patients. For example, Coffee and Gaurderer (2016) noted that both an overview for providers of existing research on nature exposure and health, and brochures tailored for patients, was provided by the National Environmental Education Foundation.

Some programs connected patients with targeted outdoor resources, such as state parks [40], or guided walks at a specific trail [44]. Third parties can provide resources such as maps to help patients locate accessible outdoor locations [40]. Many programs seek to connect patients with any outdoor resource, for example within a city or metropolitan area. In this case, providers and patients are in need of an accessible database of information to locate an outdoor space or program. In some cases, these resources do not exist. Zarr et al. (2017) described a process in which trained volunteers (e.g., physicians, park rangers, and students) conducted a survey and created a database that included descriptions and ratings of 342 parks in Washington, DC.

Funding sources reported in review papers ranged from local foundations [34,42,44], to national foundations [37,42,43,44], research institutes (including the National Institutes of Health) [40,41], hospitals [41,43], and professional societies such as the American Academy of Pediatrics [43]. Many of the programs or interventions described relied on multiple funding sources.

### 3.3. Empirical Studies: Impacts on Adherence and Health

To date, only eight empirical studies have been conducted with nature prescription patients, their caretakers, or physicians [37,38,39,40,41,42,43,44]. Five empirical studies [37,38,40,42,43] and one case study [34] focused on the health or health behavior outcomes of children and/or their families. Two studies examined health outcomes of adults; one focused on patients’ parents, and research design [35] and results [44] are described in two separate papers. The other focused on outcomes of adult cancer patients [39]. Characteristics of these studies are shown in Table 2. In addition, we describe a research design protocol for a nature prescription intervention [36].

#### 3.3.1. Studies of Patients

The target populations, outcomes measured, and results from the eight studies of patients (or their parents) varied widely and therefore few patterns can be reported. Findings on impacts to physical health and behaviors were mixed. Nature prescriptions had a positive impact on attention restoration compared to a control group among adult patients [39]. In a pre-post study of a nature prescription intervention, Christiana et al. [37] found no significant differences in parent-reported outdoor physical activity, time spent outdoors, and sedentary activities among child patients. Other studies reported an increase in reported physical activity, a decrease in stress [44], and an increase in resilience (mediated by decreased stress) [42].

A number of studies addressed the matter of adherence, either by testing program adherence directly with or without control or contrast groups, or by case study exploration. Adherence to structured prescription programs could be measured via registration and then physical presence at the staffed outings. Adherence or follow-through to unstructured nature prescriptions was measured using self-report [38,42,43,44], or by the collection of park entrance passes [40]. Coffey and Gauderer [40] was one of few studies to assess adherence rate. Participating providers gave nature prescriptions in the form of a pass to a state park. The passes, when redeemed, could be retained and counted. Authors found a redemption rate of 13% despite the fact that the wettest summer months on record for the state occurred during the study period.

Program adherence is the mechanism by which increased nature exposure, and potentially any change in health behaviors and outcomes, would occur. For example, Zarr et al. [43] examined self-reported change in physical activity attitudes and behaviors among 225 nature prescription patients. Parents were surveyed just before and three months after a physician’s visit. The authors found no significant change in parental perceptions about parks. The study did find an increase in percent of parents reporting that their child had visited a park within the past year, and that they believed physical activity affected the health of their child. Self-reported average weekly physical activity increased from 150 to 172 min, and this was accompanied by an increase in the reported number of days per month spent in a park for 30 or more minutes (from 7 to 8 days).

The studies led by Razani et al. [42,44] tested whether more intensive assistance would increase adherence by including supported and independent park prescription participant groups. Parents in both groups received physician counseling about nature, maps of local parks, a journal, and pedometers. The supported group also received phone and text reminders to attend three weekly family nature outings with free transportation, food, and programming. However, it was not clear whether the enhanced adherence support improved either adherence or health outcomes. Razani et al. [44] found that overall stress decreased between baseline and three-month follow-up in both groups, though parents in the independent group reported more park visits per week than those in the supported group. Likewise, there were no group effects on childhood resilience among participants of the same intervention [42].

Other published articles focused on the practical challenges of administering nature prescription programs and promoting adherence. One study concluded that the referral process should be easily accessible and administered by providers, and quick follow-up with patients can help keep them engaged in a nature prescription program [36]. The study also concluded that strong communication among clinical, research, and parks team members was also key to program administration [36]. Razani et al. [34] found that the success of nature prescription programs depends on the ability to tailor to the needs of individuals, families, and communities.

#### 3.3.2. Studies of Providers

Three studies [38,40,41] focused on either perspectives or outcomes associated with health care providers. These studies explored predictors of and barriers to providers in prescribing time outdoors. Study characteristics are shown in Table 2. One of these studies also included a component focusing on patients, and is listed both under patient and provider studies.

Using qualitative and survey methodologies, these studies focused on providers’ perspectives, needs, and challenges regarding participation in nature prescription programs. Providers’ lack of time was a major barrier to their participation [38,41]. Providers also desired more awareness of the benefits of parks/outdoors for health [38], better communication between themselves, program administrators and patients, and more feedback about the program’s impact [38,41].

Providers felt that patients’ lack of time, transportation and resources in general were barriers to patient participation [38], and that program attributes, such as no-/low-cost, local, fun, and potential for weight loss served as incentives to families [41].

It could be that providers’ values are a key determinant of their participation in nature prescription programs. For example, providers’ affinity for nature might increase their likelihood to write a nature prescription. However, Coffey and Gauderer [40] found no relationship between providers’ Nature Relatedness (NR) score and their likelihood to write nature prescriptions.

## 4. Discussion and Agenda for Future Research

The majority of studies we reviewed measured short-term behaviors or outcomes associated with child or adolescent patients. Except in one case [42], data was collected from the parent rather than the child. More work is needed to assess any changes in health behaviors and outcomes associated with participation in nature prescription programs for adults, for parents and family members, and for other sub-population groups. Research is also needed on the sustainability of effects. Most studies reviewed followed patients up to six months after the intervention. More research is needed to assess whether these interventions have long-lasting effects on behaviors and outcomes.

The studies reviewed incorporated a variety of methods to increase participation. First, structured programs offered programmed activities, transportation, and food. While no comparison of costs has been published, structured programs potentially require significantly greater per person costs than unstructured prescriptions. However, only two studies [42,44] tested for differences both in adherence and in health outcomes between the two formats. It will be important to assess whether, and if, these costs result in a greater effect and if the costs are commensurate with the effects.

While nature prescription programs require significant effort on the part of providers, coordinators and other actors, existing research tells us little about how to measure and increase adherence to nature prescriptions. Adherence to unstructured prescriptions, in particular, is difficult to measure; most tracked adherence via self-report, which could introduce bias. Nature prescription studies could benefit from application of other methods to track adherence, for example taking advantage of smartphones, GIS technology, and wearable sensors [49,50]. In the case of physical activity, evidence from randomized trials has shown that if medical providers formalize their counseling (through written prescriptions), adherence increases [51]. Despite calls for providers to encourage exercise counseling for over 30 years, the last decade has seen a greater emphasis of physical activity counseling integrated within a standardized clinical practice [43,52]. However, questions remain regarding the influence of counseling versus other interventions on nature prescription adherence.

In addition, while medical providers play a paramount role in nature prescription programs, we found only three studies that focused on providers. Barriers to implementation, from providers’ perspectives, were explored using qualitative, self-report methods. Further work is needed to test the effects of different tools and resources on providers’ likelihood to participate in nature prescription programs. No research to date, of which the authors are aware, has evaluated the effect of various training and education programs on provider engagement and participation. We have also not discovered research on what impacts participation in nature prescription programs has had on providers’ own health. There are key roles for other health professionals, such as nurse practitioners, nurses, social workers, and clinical staff, to play in nature prescriptions. Further research could explore the broader institutional and practical constraints and opportunities for nature prescriptions within broader health care systems.

Finally, we found few studies that evaluated program effectiveness. Our review identified only eleven articles, including eight empirical studies of such programs. Additionally, among the published studies, there were no randomized controlled trials, and all studies except Razani et al. (2018; 2019) relied on non-randomized convenience samples. While all studies collected data at multiple time points, with two exceptions [37,39], none used true control groups. In this case, it is not possible to say whether changes measured over time among study participants did not also occur in the general population.

While this literature review is the first, to date, to focus on empirical studies of clinical nature prescription interventions, it is subject to a number of limitations. Our search may have omitted relevant studies not included in the four electronic databases listed. We did not assess the included studies for bias, and we only included articles written in English.

## 5. Conclusions

Healthcare providers are increasingly looking to incorporate knowledge about environmental influences on health into therapeutic interventions. Nature prescription programs offer an opportunity to connect patients with local parks and green spaces, and to capitalize on health benefits that could result. Based on a review of relevant literature, we found that studies of nature prescription programs with a clinical component in out-patient settings focus on a wide variety of health behaviors and outcomes among mostly at-risk children and families. Research is too sparse to draw patterns in health outcome response. Formal program structure was the primary approach to increase adherence tested, however findings were mixed. Challenges and opportunities for providers is another under-explored area of research. While there is a growing number of nature prescription programs in the US and abroad, the research and evaluation of such programs overall is lacking. However, a small number of studies have set a groundwork for future research. We describe research needs in the areas of adherence, health behaviors and outcomes, and provider perspectives.

## Figures and Tables

**Table 1:** Nature prescription descriptions.

Study	Program Descriptions	Population
**Unstructured Prescriptions**		
Christiana et al. [37]	Patients receive counseling, education about local outdoor resources, and prescription for 60 min or more of outdoor physical activity per day	Children (ages 5–13)
Coffey and Gaurderer [40]	Participants receive counseling, education about local outdoor resources, and a 1-day free pass to any state park day use area	Children (ages 6–10)
Razani et al. [35,44]	Group 1: Patients receive counseling, education about local park resources, journals and pedometers	Children (low-income; ages 4–18) and parents
Razani et al. [42]	Group 1: Patients receive counseling and education about local park resources	Children (low-income; ages 7–17) and parents
Zarr et al. [43]	Patients receive education about local outdoor resources, and a prescription for outdoor physical activity	Children and adolescents (low-income)
**Structured Prescriptions**		
Cimprich and Ronis [39]	Home-based program involving 120 min of exposure to the natural environment per week	Women with newly diagnosed breast cancer
James et al. [41]	Patients receive counseling and are referred to guided outdoor activities, with incentives	Children (low-income; ages 2–13)
Messiah et al. [36]	Patients receive counseling and referral to a park-based afterschool health and wellness program	Children and adolescents (low-income; ages 6–14)
Razani et al. [34]	Patients receive counseling, and are recruited to formal outings involving unstructured nature exploration and physical activity once a month	Infants, children and adolescents (low-income; ages 0–18)
Razani et al. [35,44]	Group 2: Patients recruited to 3 nature outings to parks where they engaged in unstructured nature play, physical activity and picnic, and quiet reflection	Children (low-income; ages 4–18) and parents
Razani et al. [42]	Group 2: Three organized group outings at three parks (among the seven parks highlighted in the map given to all families) over three weeks	Children (low-income; ages 7–17) and parents

**Table 2:** Study characteristics.

Study	Target Study Population	Research Question or Aims	Target Health Outcomes	Study Design	Intervention	Methods	Results
**Patient Studies**							
Cimprich and Ronis [39]	Female patients diagnosed with breast cancer (*n* = 157)	What is the effectiveness of an early natural restorative environmental intervention aimed at counteracting attentional fatigue?	Attentional fatigue and performance, time spent in nature	Pre-post test	Patients received 120 min of home-based exposure to the natural environment per week. Control patients received no intervention.	Self-reported capacity to direct attention assessed ~17 days before and 19 days after surgery. A home-based intervention was initiated after the first assessment and before any treatment. Participants recorded type of nature activity and time spent in each activity daily.	The intervention group showed greater recovery of capacity to direct attention after therapy, compared with the nonintervention group.
Christiana et al. [37]	(1) Patients (ages 5–13 years; *n* = 38) of 2 rural providers; (2) Patients (*n* = 32) of 5 non-participating providers	What is the effectiveness and feasibility of an intervention involving health care providers talking to their patients and parents about the importance of outdoor physical activity (PA) and prescribing outdoor activity for children?	Outdoor PA, sedentary behaviors, and time spent outdoors	Longitudinal pilot study	Patients received counseling about local outdoor resources and prescription for 60 min of outdoor PA per day. Control patients received no intervention.	Surveys administered to parents at baseline, 1 and 3 months after pediatrician visit; including items from Youth Risk Behavior Surveillance System; Leisure-Time Exercise Questionnaire; amount of time doing sedentary behaviors; how much time spent outdoors; parent view of prescriptions.	Changes in children’s outdoor PA, time spent in the outdoors, and sedentary activities were not significantly different between intervention and control groups. Wald chi-square values: Days in the past week child was physically active 60 min+ anywhere (3.97) or outdoors (2.46); Frequency of PA anywhere (1.28) or outdoors (2.34); Time spent outdoors (2.99); Time spent in sedentary activity on weekdays (1.80) and on weekend days (0.80).
Coffey and Gauderer [40]	(1) Patients (ages 6–10; *n* = 1935)	(1) Does a Park Rx encourage children to engage in a nature experience in the short term, as measured by redemption of the Park Rx at a local state park?	Increased time spent in nature	Quasi-experimental pilot study	Patients received counseling, education about local outdoor resources, and a 1-day free pass to any state park day use area.	Park staff counted redeemed ParkRx passes. Families had 15 weeks to redeem.	There was a 13% redemption rate.
Messiah et al. [36]	Low-income, minority children (ages 6–14; *n* = 50) diagnosed with overweight/obesity, hypertension, or family history of diabetes and/or cardiovascular disease	Can an affordable and accessible obesity prevention and treatment program reduce childhood obesity?	Increase in physical activity, decrease in BMI	Research design	Patients received education about local outdoor resources and prescription for outdoor physical activity.	Pediatric clinics patients were enrolled in the Fit-2-Play program. Focus groups were conducted with pediatricians, park coaches, and patients.	No results provided.
Razani et al. [34]	Patients (low income; ages 0–18; *n*= 20)	Help people engage in nature by diminishing the barriers (transportation, food, child care needs)	Combat stress and build resilience	Field report	Patients received counseling, and outings involving unstructured nature exploration and physical activity once a month.	Observation	Nature was a tool to deal with stressors associated with poverty; stress relief and time to relax with family motivated participation more than physical activity; variations in temperament and developmental stage make each child’s response unique; parents’ efforts to get their children outdoors should be acknowledged; being culturally responsive is important in nature.
Razani et al. [44]	Parents of patients (low income; ages 4–18; *n* = 78)	(1) Do park prescriptions improve parents’ stress, park visits, loneliness, physical activity and nature affinity? (2) Will a supported park prescription have a greater impact on stress and other outcomes than an unsupported prescription?	Physical activity, stress, loneliness, park visits per week	Randomized clinical trial with pre-post survey	Group 1: Patients received counseling, education about local park resources, journal and pedometer. Group 2: Patients recruited to 3 park outings where they engaged in unstructured nature play, physical activity and picnic, and quiet reflection.	Measures included Perceived Stress Scale, park visits, step counts, physical activity, UCLA Loneliness Score, salivary cortisol, and nature affinity. Measures occurred in both groups at 0, 1, and 3 months after enrollment.	Both groups saw decreases in stress (1.71 points); loneliness (1.03 points); cortisol level (0.05 μg/dL); and increases in park visits (1.22 visits per week); in time spent in moderate physical activity per week (24 min); and nature affinity (0.35 points). The unsupported group had a significant increase park visits compared to the supported group.
Razani et al. [42]	Patients (low-income; ages 7–17; *n*= 54) and their parent	(1) Are park visits associated with pediatric resilience over the three months after patients received a park prescription? (2) Are pediatric stress levels a mediating factor between weekly park visits and resilience?	Resilience, stress, park visits per week	Prospective longitudinal clinical trial	Patients recruited to 3 park outings over 3 weeks and received counseling. Patients assigned to intervention group received support in getting to the parks.	Parents reported their child’s park visits per week, baseline adverse childhood experience score, their own stress and coping; children reported resilience and stress. Measures occurred in both groups at 0, 1, and 3 months after enrollment.	Resilience improved with each 1-day increase in weekly park visits (0.04 points (0.01, 0.08) at every ACEs level.
Zarr et al. [43]	Patients (low-income; child & adolescent; *n* = 225 families) at risk for chronic illnesses	What is the impact of provider-based park prescriptions on outdoor physical activity?	Physical activity	Pre-post survey	Patients received education about local outdoor resources and prescription for outdoor physical activity.	Surveys administered to parents immediately before and 3 months after the intervention to assess changes in attitudes and behaviors around physical activity.	No significant changes in parental perceptions about parks or physical activity. Significant increase in the proportion of parents reporting of child’s park visits in the past year and that they believed that physical activity affected their child’s health.
**Provider Studies**							
Christiana et al. [38]	Children’s health care providers (*n* = 15)	What are the barriers for health care providers to prescribing outdoor physical activity?	Physician perspective	Interviews	None	Semi-structured interviews to explore perspectives on outdoor PA prescription programs for children and barriers to implementation.	Providers’ lack of time, awareness of the benefits of parks/outdoors, and of programs’ effectiveness, and perceived patient barriers, were major barriers to program participation.
Coffey and Gauderer [40]	(2) Primary care providers (PCPs; *n* = 24)	(1) Does the PCP’s NR and/or participation in the pilot impact their likelihood of writing a park prescription? (2) Did study participation impact the PCP’s likelihood of discussing the value of nature during a well-child exam?	PCP Nature Relatedness (NR) score; likelihood to discuss the importance of nature during well-child exam	Quasi-experimental pilot study	Patients received counseling, education about local outdoor resources, and a one-day free pass to any state park day use area.	Surveyed providers about nature relatedness and likelihood to discuss nature with patients.	There were no difference in rate of park prescription between providers with low and high NR scores. Participation in the program increased PCP promotion of nature experiences.
James et al. [41]	Pediatricians participating in Outdoors Rx program (*n* = 23)	How do pediatricians view the utility of Outdoors Rx, barriers to success, and opportunities for improvement?	Pediatrician perspective	One-time survey	Providers gave patients counseling and referred them to guided outdoor activities, with incentives.	Surveyed pediatricians on Outdoors Rx: (a) referral patterns (b) impact on physical activity counseling, (c) perceived patient interest (d) barriers to success, and (e) suggestions for improvement.	Findings reveal providers’ referral patterns, participation impact on physical activity counseling, perceived patient interest, barriers to success, and suggestions for improvement.

## Supplementary Materials

The following are available online at https://www.mdpi.com/1660-4601/17/12/4213/s1, Database Search Terms.
