# Supplementary material for: Nature Prescriptions for Health: A Review of Evidence and Research Opportunities
Source: Int J Environ Res Public Health. 2020 Jun 12;17(12):4213. doi: 10.3390/ijerph17124213 (PMC7344564; doi:10.3390/ijerph17124213)
Supplement: Supplementary file 1 [file ijerph-17-04213-s001.pdf]

Supplementary Material

Database Search Terms

Scopus
“outdoor activity prescription*” OR “nature prescription*” OR “park prescription*” OR “outdoor prescription*” OR “prescrib* nature” OR “outdoor play” OR “nature based activit*” OR “outdoor physical activity” OR “nature engagement” OR “green prescription*” OR “outdoor prescription*” OR “green exercise” OR “nature play” OR “nature AND outdoor activit*” OR “green care” OR “wander garden therap*” OR “ecotherapy*” OR “horticultural therap*” OR “nature assisted therap*”

EMBASE
‘outdoor activity prescription*’ OR ‘nature prescription*’ OR ‘park prescription*’ OR ‘outdoor prescription*’ OR ‘prescrib* nature’ OR ‘outdoor play’ OR ‘nature based activit*’ OR ‘outdoor physical activity’ OR ‘nature engagement’ OR ‘green prescription*’ OR ‘outdoor prescription*’ OR ‘green exercise’ OR ‘nature play’ OR ‘nature AND outdoor activit*’ OR ‘green care’ OR ‘wander garden therap*’ OR ‘ecotherapy*’ OR ‘horticultural therap*’ OR ‘nature assisted therap*’

MEDLINE
outdoor activity prescription OR nature prescription OR park prescription OR outdoor prescription OR prescribe* nature OR outdoor play OR nature-based activity OR outdoor physical activity OR nature engagement OR green prescription OR outdoor prescription OR green exercise OR nature play OR nature AND outdo activit* OR green care OR wander garden therap* OR ecotherapy OR horticultural therap OR nature assisted therap*
